# The Effects of Pre-Operative Enteral Nutrition from Nasal Feeding Tubes on Gastric Outlet Obstruction

**DOI:** 10.3390/nu9040373

**Published:** 2017-04-10

**Authors:** Zhi-hua Chen, Su-yong Lin, Qi-bao Dai, Jin Hua, Shao-qin Chen

**Affiliations:** Department of Gastrointestinal Surgery, The First Affiliated Hospital of Fujian Medical University, Fuzhou 350005, China; chenzhihua198613@sina.com (Z.C.); fyyyczh@sina.com (S.L.); daijin@yahoo.com (Q.D.); ribo191@163.com (J.H.)

**Keywords:** pre-operative, enteral nutrition, gastric outlet obstruction, gastroscope, immune function

## Abstract

We examined gastric outlet obstruction (GOO) patients who received two weeks of strengthening pre-operative enteral nutrition therapy (pre-EN) through a nasal–jejenal feeding tube placed under a gastroscope to evaluate the feasibility and potential benefit of pre-EN compared to parenteral nutrition (PN). In this study, 68 patients confirmed to have GOO with upper-gastrointestinal contrast and who accepted the operation were randomized into an EN group and a PN group. The differences in nutritional status, immune function, post-operative complications, weight of patients, first bowel sound and first flatus time, pull tube time, length of hospital stay (LOH), and cost of hospitalization between pre-operation and post-operation were all recorded. Statistical analyses were performed using the chi square test and *t*-test; statistical significance was defined as *p* < 0.05. The success rate of the placement was 91.18% (three out of 31 cases). After pre-EN, the levels of weight, albumin (ALB), prealbumin (PA), and transferrin (TNF) in the EN group were significantly increased by pre-operation day compared to admission day, but were not significantly increased in the PN group; the weights in the EN group were significantly increased compared to the PN group by pre-operation day and day of discharge; total protein (TP), ALB, PA, and TNF of the EN group were significantly increased compared to the PN group on pre-operation and post-operative days one and three. The levels of CD3+, CD4+/CD8+, IgA, and IgM in the EN group were higher than those of the PN group at pre-operation and post-operation; the EN group had a significantly lower incidence of poor wound healing, peritoneal cavity infection, pneumonia, and a shorter first bowel sound time, first flatus time, and post-operation hospital stay than the PN group. Pre-EN through a nasal–jejunum feeding tube and placed under a gastroscope in GOO patients was safe, feasible, and beneficial to the nutrition status, immune function, and gastrointestinal function, and sped up recovery, while not increasing the cost of hospitalization.

## 1. Introduction

Gastric outlet obstruction (GOO) is a mechanical gastric emptying dysfunction which is mainly caused by a distal gastric cancer invasion helicobacter tube and scarring after gastroduodenal ulcer healing [1]. Abdominal distension and vomiting are the main clinical manifestations, with incidences of up to 50% [2]. As the disease progresses, vomiting and abdominal distension are increasingly severe, and result in eating problems, which ultimately result in dystrophy, water and electrolyte balance disorders [3], and an increase in stomach edema and the degree of gastric outlet obstruction. Often, coupled with this are long-term nutrient consumption problems, caused by most types of gastric cancer; thus, the vast majority of GOO patients have different degrees of malnutrition. Removal of the obstruction is the primary goal in the treatment of GOO patients, and the main method of treatment is surgery [4]. However, edema of the stomach and malnutrition lead to a decrease in the immune system, which increases the risk of peri-operative infection, poor healing of anastomotic and incision sites, and poor post-operative recovery, which also increases the risk of peri-operative pulmonary infection due to vomiting, causing aspiration into the lungs of patients with GOO [3]. In short, the peri-operative malnutrition of GOO not only increases the length of hospital stay and hospital costs, but also delays the best opportunity for gastric cancer patients with GOO to receive post-operative chemotherapy. Finally, it prevents patients with GOO from getting the best results from treatment, and has a negative impact on their quality of life [5]. Thus, we decided to strengthen pre-operative enteral nutrition therapy by means of a nutritional tube under a gastroscope to verify the feasibility and potential benefits of pre-operative enteral nutrition for patients with GOO in the post-operative course.

## 2. Patients and Methods

### 2.1. Patient Selection

We recruited 68 subjects from the Department of Gastrointestinal Surgery of the First Affiliated Hospital of Fujian Medical University between January 2014 and December 2016, all confirmed to have gastric outlet obstruction with upper gastrointestinal contrast and gastroscopy. The inclusion and exclusion criteria were: (1) aged between 18 and 80; (2) the focal disease could be cured surgically; (3) patients had not received pre-operative radiation or chemotherapy treatments; (4) no serious liver, kidney, heart, or lung function insufficiencies in cases where patients could not tolerate surgery; (5) cases where surgery was refused were excluded; and (6) all patients were randomized into an enteral nutrition group (EN group) and a parenteral nutrition group (PN group). In the EN group, patients had a nasal–jejunum feeding tube with three channels, and at the same time had a decompression tube placed under a gastroscope. The study had been approved by the Ethics Committee of the First Affiliated Hospital of Fujian Medical University, and informed consent was obtained according to the Declaration of Helsinki (No. 2014-0124R).

### 2.2. Methods

The total daily food intake for each patient was required to be at least 146.50 kJ (35 kcal)/kg. The enteral nutrient solution (Nutrison) was fed through a tube before surgery in the EN group, and the residual energy was made up by infusing a fat emulsion, amino acids (17), and an injection containing 1% glucose (1440 mL/bag; Sino-Swed Pharmaceutical Corp, Wuxi, China). A total of 500 mL of nutrient solution was given on the first day, and the dose was increased by 500 mL every 24 h, provided that there were no problems related to EN. Once reached, the EN maximum daily dose of 1500–2000 mL was maintained for 14 days before surgery. The total energy of the PN group was attained by infusing the total parenteral nutrition (TPN) all in one bag, and was maintained for no longer than 7 days before the surgery. All patients had a decompression tube before the operation, which was fed into the intestine across the anastomotic stoma during the operation, and were given sequential nutrition support after the operation. All energy was initially provided by TPN, and was gradually transformed into total enteral nutrition, according to gastrointestinal function recovery in all groups, and a required total daily calorie intake of 146.50 kJ (35 kcal)/kg.

### 2.3. Clinical Assessment

Clinical factors included age, gender, weight, smoking, drinking habits, diabetes, lesion (benign mechanical or gastric cancer), nutritional risk screening according to the NRS-2002 of the European Society of Parenteral Enteral Nutrition (ESPEN) [6], modus operandi, and digestive tract reconstruction. The length of hospital stay (LOH), length of post-operation hospital stay (PO-LOH), number of uses of albumin, first bowel sound time (expressed as hours), first flatus time (expressed as hours), pull tube time (expressed as days), and cost of hospitalization were also recorded. Peripheral blood was collected on admission day (AD-D), pre-operative day (Pre-Op), and the first, third, and seventh post-operative (POD) days. The nutritional statuses were expressed by the levels of hemachrome (HB), total protein, albumin (ALB), prealbumin (PA), and transferrin (TNF), and immune function was determined using the levels of total lymphocytes (Lym), CD3+, CD4+/CD8+, IgA, IgM, and IgG. Post-operative complications, including pneumonia, acute respiratory distress syndrome (ARDS), peritoneal cavity infection, anastomotic fistula, poor wound healing, severe abdominal distension, and functional delayed gastric emptying, were also recorded.

### 2.4. Statistical Analyses

All analyses were carried out using the SPSS 19.0 software package (IBM, New York, NY, USA), and measurement data were indicated as mean standard deviation. Qualitative data were analyzed using the chi-square test; examined measurement data were analyzed using the *t*-test, and *p*-value < 0.05 was considered significant for statistical analyses.

## 3. Results

### 3.1. Peri-Operative Clinical Features

Cases where the tube placement failed were included in the PN group (three cases in total), and the success rate of the placement was 91.18%. There were 31 cases in the EN group and 37 cases in the PN group. There was no significant difference in the mean age, gender, lesion, pre-operative nutritional risk screening, smoking, drinking habits, diabetes, modus operandi, and digestive tract reconstruction between the two groups (Table 1).

### 3.2. Pre-Operative Nutritional Treatment

In the pre-operative nutritional treatment, the total daily calories of the two groups were not significantly different (*p* = 0.185). The nutritional treatment time of the PN group was significantly shorter than that of the EN group (*p* < 0.05), but the cost of daily nutrition for the EN group was significantly lower, and the total cost of nutrition of the EN group was also lower than the PN group (*p* < 0.05) (Table 2).

### 3.3. Peri-Operative Nutritional Index

The weights of the two groups were not significantly different on admission day (AD-D) (*p* = 0.405). After strengthening the pre-operative enteral nutrition support, the weight of the EN group was significantly increased at Pre-Op compared to AD-D (*p* = 0.002), but not significantly increased in the PN group (*p* = 0.341); the weight of the EN group was significantly higher than the PN group at Pre-Op and the day of discharge (*p* < 0.05) (Table 3).

The hemachrome (HB), TP, ALB, PA, and TNF of the two groups was not significantly different on the AD-D (*p* > 0.05). After the pre-EN support, the HB, ALB, PA, and TNF of the EN group was significantly increased on Pre-Op compared to AD-D (*p* < 0.05); however, the HBs of the two groups were not significantly different (*p* > 0.05). The HB, ALB, PA, and TNF of the EN group were significantly increased compared to the PN group at Pre-Op and on POD 1and 3 (*p* < 0.05), but there was no significant difference between the two groups at POD 7 (*p* > 0.05). HB was not significantly different between the two groups at Pre-Op or on POD 1, 3, and 7 (*p* > 0.05) (Table 4).

### 3.4. Peri-Operative Immune Index

The total level of the two groups’ immune functions was not significantly different on AD-D (*p* > 0.05). After pre-EN support, the total levels of immune function of the EN group were significantly increased at Pre-Op compared to AD-D (*p* < 0.05), but were not significantly different in the PN group (*p* > 0.05). The Lym level in the EN group was significantly increased compared to the PN group at Pre-Op; CD3+ level was increased at Pre-Op and POD1; CD4+/CD8+ levels were increased at Pre-Op; IgA levels were increased at Pre-Op and POD1, 3, and 7; and IgM was increased at Pre-Op and POD1 and 3 (*p* < 0.05). The IgG level was not significantly different between the two groups at Pre-Op or POD1, 3, and 7 (*p* > 0.05) (Table 5).

### 3.5. Post-Operative Gastrointestinal Function Recovery, Complications, and Mortality

Patients in the EN group had a significantly shorter first bowel sound time and first flatus time than the PN group (*p* < 0.05); however, the pull tube time was similar in the two groups (*p* > 0.05) (Table 6).

Post-operative complications such as poor wound healing, peritoneal cavity infection, and pneumonia were significantly different between the two groups (*p* < 0.05). The incidences of anastomotic fistula, functionally-delayed gastric emptying, and ARDS of the PN group were higher than the EN group, but the differences were not statistically significant (*p* > 0.05). There was only one death in the PN group; the mortality rate was not statistically significant between the two groups (*p* > 0.05) (Table 7).

### 3.6. Post-Operative Outcomes

There were more cases requiring the use of albumin in the PN group (15 out of 37) than the EN group (5 out of 31, *p* < 0.05). The length of hospital stay in the EN group was longer than that of the PN group (*p* < 0.05); however, the length of post-operation hospital stay for the EN group was shorter than that of the PN group (*p* < 0.05). The difference in the total hospitalization costs of the two groups was not statistically significant (*p* > 0.05) (Table 8).

## 4. Discussion

Gastric outlet obstruction is caused by mechanical gastroduodenal obstruction or motility disorders, which are divided into three groups: benign mechanical, malignant mechanical, and motility disorder [1]. Peptic ulcer disease is the most common cause of benign mechanical GOO, and the clinical incidence of benign mechanical GOO has decreased along with the decline of peptic ulcer disease in recent years [7]. Malignant mechanical GOO usually results from cancer—primarily gastric or pancreatic, affecting the distal stomach or proximal duodenum. Most GOOs are the result of gastric carcinoma, and the incidence of gastric cancer is ranked as the second most common malignant tumor in China. Patients with GOO may present with nausea and vomiting, weight loss, abdominal bloating, early satiety, and/or abdominal discomfort. Abdominal distension and vomiting are the main symptoms, and have an incidence rate of up to 47% [2]. Persistent vomiting, resulting in an excessive loss of digestive juices and food, difficulty digesting food in the stomach, and difficulty absorbing nutrients in the intestines, can lead to dehydration and electrolyte imbalance in the body; therefore, patients are vulnerable to malnutrition as well as water and electrolyte balance disorders [3]. The incidence of malnutrition is as high as 40%–80% in patients with distal gastric malignant tumors who are chronically undergoing tumor nutrition digestion [8]. As such, most peri-operative GOO patients have different degrees of malnutrition. There were 49 cases with risk of the nutrient in total, and the rate was up to 72.06%, as evaluated in this research using the NRS-2002 system (2002, European Society Of Parenteral Enteralnutrition, Germany).

Surgery is an important intervention in the treatment of gastric cancer with GOO [4], and the effects of this treatment are ideal, with symptom remission rates as high as 72% [9]. For patients undergoing surgery, pre-operative nutritional condition directly affects post-operative prognosis, overall survival, and disease-specific survival [10]. Malnutrition of patients not only causes weakening of muscle activity and delayed healing of incisions [11], but may also cause a drop in the body’s immune system functions, resulting in neutrophils, macrophages, and lymphocyte function abatement, and increasing the occurrence of peri-operative infections such as pulmonary infection and abdominal cavity infection. Therefore, malnutrition can increase post-operative complications, be unfavorable for post-operative recovery and the length of hospital stay, and also indirectly increase a patient’s psychological and economic burdens, seriously affecting the patient’s quality of life [12]. Liu found that pre-operative nutritional status (PNS) was a valuable predictor of outcomes and was independently associated with overall survival (OS) rates in patients with gastric cancer [13]. Additionally, some reports show that the direct cause of death in more than 20% of tumor patients is malnutrition [14]. As such, most patients with GOO need peri-operative nutritional support treatment.

Enteral nutrition can support patients with a variety of amino acid digestion and absorption problems, and can also improve protein utilization to accelerate wound healing [15], activate the gastrointestinal nerve endocrine system, promote intestinal peristalsis, accelerate nutrient absorption, improve intestinal physiological and ecological balance, and protect the intestinal mucosa so as to maintain normal gastrointestinal tract functions and avoid intestinal mucosa atrophy and intestinal bacterial translocation, which cause infections. At the same time, enteral nutrition preparations containing many kinds of necessary nutrients can provide the body with a comprehensive nutrition, restrain inflammation, improve immunity, and reduce the peri-operative incidence of infectious complications [16]. Enteral nutrition therapy is safe, effective, economical, and convenient [17], and has a wide range of applications in clinics. It is generally believed that as long as the function of the intestines remains intact, the early use of enteral nutrition support should, as far as possible, be used [18].

With the development of endoscopic techniques and the technology of endoscopic placement, nasal–jejunum feeding tubes now have common clinical applications and continue to be improved [18]. This not only provides relatively safe management for patients with upper gastrointestinal obstructions, but also reduces the need for parenteral nutrition treatment [19]. A nasal–jejunum nutrition tube through the digestive tract stenosis site, guided using a gastroscope, will deliver nutrients directly to the jejunum, and is especially suitable for patients with GOO. This study established the value of endoscopic placement of nasal–jejunum feeding tubes in patients with GOO, and the success rate of placement was 91.18%. We also found that enteral nutrition was safe and well-tolerated by peri-operative GOO patients. At present, most of the research that focuses on early post-operative EN therapy has verified that early EN can improve the nutritional status and immune the function of patients with gastric cancer, help regulate the post-operative response of patients after gastric cancer surgery, promote rehabilitation, accelerate the recovery of gastrointestinal function, and promote the early recovery of intestinal function [20]; furthermore, it is inexpensive. Ding found that the one week of pre-operative EN support improved post-operative nutritional status and immune function, alleviated inflammatory response, and facilitated recovery for gastric cancer patients [21]. We found that after two weeks of pre-operative strengthening EN treatment (which prolonged the preoperative nutritional treatment time), the pre-operative nutritional indices were significantly higher than those of the control group, including those that may also be caused by prolonged nutritional treatment (e.g., the mean weight of patients, ALB, PA, and TFN), so the patients acquire better nutritional reserves from ample and long-term enteral nutrition. After surgical trauma and nutrition digestion, the post-operative nutritional indices of the two groups on the first post-operative day were significantly reduced, but the post-operation nutrition and immune indices of the EN group were still higher than in the control group, as pre-operative strengthening nutrition support therapy was able to correct part of the pre-operative malnutrition and provide more peri-operative nutrition reserves, while using less albumin. EN therapy could also preserve the function of the small intestine, promote the recovery of gastrointestinal function, and reduce bacteria that could lead to ectopic peri-operative infection. Therefore, the pre-operative strengthening EN treatment was obviously able to increase peri-operative immunity and reduce peri-operative intraperitoneal infection, poor healing of incisions, and the occurrence of complications of lung infection, and also give patients the opportunity for a better recovery, reducing their post-operative hospital stay.

In China, hospital management, medical insurance management centers, and the government strictly control the length of stay and hospitalization expenses; therefore, clinical doctors are always afraid of pre-operative enteral nutrition support treatment, which can increase total hospitalization expenses and hospitalization times, thus they do not consider it an option. However, we found that pre-operative EN treatment was safe and effective. Although we increased the time of preoperative nutrition treatment, we also found that the total cost of nutrition for the EN group was also lower than that of the PN group because of the low cost of enteral nutrient solution. The post-operative hospital stay for the EN group was reduced by an average of three days in this study, which offsets some of the increased the pre-operative hospital stay costs, and pre-operative EN treatment could not only reduce post-operative complications and the albumin used, but also would not increase the total hospitalization expenses for patients. It has been verified that early post-operative enteral nutrition therapy can reduce the total cost of hospitalizations [22]. In short, patients would benefit the most from pre-operative EN therapy, and long-term preoperative enteral nutrition therapy is very valuable, although it increases the number of days for pre-operative hospital stay. It is worth encouraging this treatment, which could improve clinical efficacy for patients.

## 5. Conclusions

Two weeks of pre-operative strengthening EN treatment is safe and effective for post-operative gastric outlet obstruction patients, and has advantages in improving post-operative nutrition status and immunological function. Although it increased the number of days needed for pre-operative hospital stay to strengthen EN treatment, this consequently reduced the incidences of peri-operative pulmonary infection, intraperitoneal infection, poor healing of incisions, promoted early recovery of intestinal movement, and reduced post-operative hospital stay, while not increasing the cost of hospitalization. Patients can also expect a better recovery.

## Figures and Tables

**Table 1:** The peri-operative clinical features of the two groups.

Variable		EN Group (*n* = 31)	PN Group (*n* = 37)	*p*
Gender (%)	Male	19	21	0.705
	Female	12	16	
Age mean (SD) year		48.6 ± 12.5	52.1 ± 13.2	0.422
Lesion	Cicatricial	7	8	0.924
	Gastric Cancer	24	29	
Nutritional risk screening	Yes	21	28	0.95
	No	10	9	
BMI		20.46 ± 2.86	21.58 ± 3.13	0.613
Smoking status (%)	Yes	12	15	0.875
	No	19	22	
Drinking (%)	Yes	7	13	0.258
	No	24	24	
Diabetes (%)	Yes	28	33	0.878
	No	3	4	
Modus operandi (%)	Laparotomy	26	28	0.405
	Laparoscopic	5	9	
Digestive tract reconstruction (%)	Billroth I	9	9	0.662
	Billroth II	15	16	
	Roux-en-Y	7	12	
Cancer staging (%)	I and II	5	6	0.709
	III and IV	19	23	

EN: enteral nutrition; PN: parenteral nutrition; SD: standard deviation.

**Table 2:** The pre-operative nutritional treatment of the two groups.

Group	Total Calorie (KJ/kg·Day)	Nutritional Treatment Time (Day)	Cost of Daily Nutrition (RMB/Day, ¥)	Total Cost of Nutrition (RMB, ¥)
EN	161.22 ± 15.45	14.61 ± 1.76	162.51 ± 21.73	2579.62 ± 173.28
PN	154.43 ± 8.31	6.73 ± 2.73	483.84 ± 36.45	3362.71 ± 284.56
*p*	0.185	0.001 ^#^	0.001 ^#^	0.023 ^#^

^#^: *p* < 0.05.

**Table 3:** The peri-operative weight of the two groups.

Group	AD-D (kg)	Pre-Op (kg)	Discharge Day (kg)
EN	53.97 ± 7.45	56.35 ± 6.67	52.23 ± 5.49
PN	52.33 ± 6.06	52.81 ± 5.41	48.97 ± 4.26
*p*	0.405	0.046 ^#^	0.035 ^#^

AD-D: admission day; Pre-Op: pre-operation; ^#^: *p* < 0.05.

**Table 4:** The peri-operative nutritional indices of the two groups.

TEST Items	Group	AD-D	Pre-Op	POD1	POD3	POD7
HB (g/L)	EN	106.9 ± 18.66	110.2 ± 16.13	99.33 ± 13.91	103.43 ± 11.56	108.7 ± 13.56
	PN	105.03 ± 13.60	104.76 ± 13.12	93.62 ± 9.95	98.68 ± 8.95	106.18 ± 9.17
	*p*	0.69	0.051	0.104	0.361	0.533
ALB (g/L)	EN	30.1 ± 3.92	36.74 ± 3.11 *	31.56 ± 2.85	32.42 ± 3.36	33.82 ± 3.05
	PN	31.37 ± 3.39	30.98 ± 3.76	22.14 ± 2.26	26.58 ± 3.61	32.34 ± 3.47
	*p*	0.233	0.027 ^#^	0.009 ^#^	0.037 ^#^	0.169
TP (g/L)	EN	53.45 ± 4.78	58.38 ± 6.11 *	55.06 ± 5.51	56.32 ± 4.84	59.81 ± 5.13
	PN	54.83 ± 5.23	53.62 ± 5.72	48.79 ± 4.06	51.88 ± 4.28	57.27 ± 5.62
	*p*	0.412	0.013 ^#^	0.001 ^#^	0.041 ^#^	0.484
PA (g/L)	EN	0.19 ± 0.018	0.27 ± 0.023 *	0.22 ± 0.017	0.25 ± 0.020	0.27 ± 0.021
	PN	0.20 ± 0.019	0.19 ± 0.019	0.15 ± 0.011	0.21 ± 0.019	0.25 ± 0.019
	*p*	0.185	0.001	0.001	0.017	0.056
TFN (g/L)	EN	1.76 ± 0.25	1.97 ± 0.28 *	1.88 ± 0.20	1.92 ± 0.23	1.94 ± 0.21
	PN	1.83 ± 0.33	1.64 ± 0.27 *	1.57 ± 0.17	1.61 ± 0.19	1.66 ± 0.23
	*p*	0.276	0.003 ^#^	0.007 ^#^	0.021 ^#^	0.018 ^#^

AD-D: admission day; Pre-Op: pre-operation; POD: post-operative day; HB: hemachrome; TP: total protein; ALB: albumin; PA: prealbumin; TFN: transferrin; ^#^: *p* < 0.05 vs. PN group; *: *p* < 0.05 vs. at AD-D.

**Table 5:** The peri-operative nutritional index of the two groups.

TEST Items	Group	AD-D	Pre-Op	POD1	POD3	POD7
Lym (×10^9^/L)	EN	1.022 ± 0.14	1.242 ± 0.12 *	1.04 ± 0.12	1.12 ± 0.12	1.28 ± 0.15
	PN	1.117 ± 0.13	1.07 ± 0.13	0.89 ± 0.11	1.08 ± 0.13	1.18 ± 0.13
	*p*	0.41	0.035 ^#^	0.068	0.164	0.381
CD3+ (%)	EN	58.83 ± 3.11	63.38 ± 6.51 *	57.38 ± 4.18	61.21 ± 5.51	61.78 ± 4.26
	PN	59.72 ± 4.42	57.35 ± 4.29	53.41 ± 2.51	57.82 ± 5.38	59.23 ± 5.11
	*p*	0.231	0.018 ^#^	0.037 ^#^	0.055	0.423
CD4+/CD8+	EN	1.49 ± 0.41	1.75 ± 0.38 *	1.52 ± 0.33	1.55 ± 0.24	1.62 ± 0.33
	PN	1.51 ± 0.53	1.32 ± 0.56	1.17 ± 0.48	1.42 ± 0.37	1.57 ± 0.25
	*p*	0.634	0.042 ^#^	0.053	0.412	0.791
IgA (g/L)	EN	1.88 ± 0.42	2.42 ± 0.63 *	2.01 ± 0.73	2.13 ± 0.53	2.29 ± 0.45
	PN	1.93 ± 0.51	1.73 ± 0.38	1.59 ± 0.68	1.61 ± 0.48	1.86 ± 0.43
	*p*	0.812	0.029 ^#^	0.036 ^#^	0.027 ^#^	0.021 ^#^
IgM (g/L)	EN	1.05 ± 0.20	1.33 ± 0.53 *	1.18 ± 0.28	1.13 ± 0.34	1.19 ± 0.57
	PN	1.12 ± 0.36	0.95 ± 0.69	0.88 ± 0.32	0.92 ± 0.21	1.08 ± 0.28
	*p*	0.599	0.008 ^#^	0.023 ^#^	0.041 ^#^	0.341
IgG (g/L)	EN	9.623 ± 1.424	11.137 ± 1.539 *	9.974 ± 1.462	9.851 ± 1.371	10.823 ± 1.149
	PN	9.837 ± 1.581	10.05 ± 1.602	9.217 ± 1.361	9.332 ± 1.425	9.842 ± 1.341
	*p*	0.625	0.059	0.276	0.667	0.572

Lym: total lymphocytes; AD-D: admission day; Pre-Op: pre-operation; POD: post-operative day; ^#^: *p* < 0.05 vs. PN group; *: *p* < 0.05 vs. at AD-D.

**Table 6:** The post-operative gastrointestinal function recovery of the two groups.

Group	First Bowel Sound Time (h)	First Flatus Time (h)	Pull Tube Time (Day)
EN	40.667 ± 9.13	64.0 ± 10.95	3.476 ± 0.68
PN	47.778 ± 9.39	74.667 ± 15.72	3.703 ± 0.72
*p*	0.011 ^#^	0.012 ^#^	0.273

^#^: *p* < 0.05.

**Table 7:** The post-operative complications and mortality of the two groups.

Group	Poor Wound Healing	Peritoneal Cavity Infection	Anastomotic Fistula	Pneumonia	ARDS (Acute Respiratory Distress Syndrome)	Functional Delayed Gastric Emptying
EN	3	2	1	2	0	1
PN	11	9	5	9	2	4
*p*	0.042 ^#^	0.046 ^#^	0.136	0.046 ^#^	0.192	0.236

^#^: *p* < 0.05.

**Table 8:** The post-operative outcomes of the two groups.

Group	Case of the Use of Albumin	LOH (d)	PO-LOH (d)	Total Cost (RMB, ¥)
EN	5	22.57 ± 2.37	8.52 ± 2.36	61,618.2 ± 10,390.2
PN	15	20.07 ± 3.27	11.96 ± 4.93	62,059.3 ± 11,004.4
*p*	0.028 ^#^	0.005 ^#^	0.001 ^#^	0.369

LOH: Length of hospital stay; PO-LOH: Length of post-operation hospital stay; ^#^: *p* < 0.05.
